# A Nomogram Model Identifies Eosinophilic Frequencies to Powerfully Discriminate Kawasaki Disease From Febrile Infections

**DOI:** 10.3389/fped.2020.559389

**Published:** 2020-12-11

**Authors:** Xiao-Ping Liu, Yi-Shuang Huang, Han-Bing Xia, Yi Sun, Xin-Ling Lang, Qiang-Zi Li, Chun-Yi Liu, Ho-Chang Kuo, Wei-Dong Huang, Xi Liu

**Affiliations:** ^1^The Department of Emergency and Pediatrics, Shenzhen Baoan Women's and Children's Hospital, Jinan University, Shenzhen, China; ^2^Department of Pediatrics, Kawasaki Disease Center, Kaohsiung Chang Gung Memorial Hospital, Kaohsiung, Taiwan; ^3^College of Medicine, Chang Gung University, Taoyuan, Taiwan

**Keywords:** Kawasaki disease, nomogram model, white blood cell (WBC), alanine transaminase (ALT), eosinophil, albumin (ALB), C-reactive protein

## Abstract

**Background:** Kawasaki disease (KD) is a form of systemic vasculitis that occurs primarily in children under the age of 5 years old. No single laboratory data can currently distinguish KD from other febrile infection diseases. The purpose of this study was to establish a laboratory data model that can differentiate between KD and other febrile diseases caused by an infection in order to prevent coronary artery complications in KD.

**Methods:** This study consisted of a total of 800 children (249 KD and 551 age- and gender-matched non-KD febrile infection illness) as a case-control study. Laboratory findings were analyzed using univariable, multivariable logistic regression, and nomogram models.

**Results:** We selected 562 children at random as the model group and 238 as the validation group. The predictive nomogram included high eosinophil percentage (100 points), high C-reactive protein (93 points), high alanine transaminase (84 points), low albumin (79 points), and high white blood cell (64 points), which generated an area under the curve of 0.873 for the model group and 0.905 for the validation group. Eosinophilia showed the highest OR: 5.015 (95% CI:−3.068–8.197) during multiple logistic regression. The sensitivity and specificity in the validation group were 84.1 and 86%, respectively. The calibration curves of the validation group for the probability of KD showed near an agreement to the actual probability.

**Conclusion:** Eosinophilia is a major factor in this nomogram model and had high precision for predicting KD. This report is the first among the existing literature to demonstrate the important role of eosinophil in KD by nomogram.

## Background

Kawasaki disease (KD) is a form of systemic vasculitis that primarily occurs in children under the age of 5 years old. The disease mainly affects small and medium-sized arteries, with coronary arteries being the most significant. Recent epidemiological investigations have indicated that the incidence of this disease is increasing every year (1). Furthermore, KD has become the main cause of acquired cardiovascular diseases in children, thus attracting even more attention. Since both the etiology and underlying mechanism of KD remain unknown, KD is typically diagnosed using clinical manifestations and laboratory findings, which may increase the rate of misdiagnosis (2). Studies have reported that ~20–25% of KD children that are untreated develop coronary aneurysms (CAA), but intravenous immunoglobulin (IVIG) [2g/(kg/day)] can reduce the incidence of CAA to 3–5% (3). Coronary artery dilation has been found in approximately 30% of KD patients in the acute stage, but mostly in the transient form (4). Therefore, establishing an effective early differentiation method and scheme is of great clinical significance for the proper diagnosis and treatment of KD.

From a review of the literature, most predictive score systems are aimed at IVIG resistance (white blood cell, albumin, C reactive protein, sodium, neutrophil, lymphocyte, eosinophilia, total bilirubin, platelet, and red blood cell distribution width, etc.) (5) or coronary artery lesions (T help 2 cytokines, albumin, Tenascin-C, monocytes, and eosinophils, etc.) (6). Few studies have focused on a predictive score for differentiating KD from other febrile infection illnesses (7). Therefore, in this study, we aimed to establish a scoring system using only clinical laboratory data to differentiate KD patients from other infection illnesses with fever. Using laboratory data instead of clinical symptoms and signs will decrease the subjectivity of the results.

A nomogram is a visualization of complex mathematical formulas resulting from traditional statistical methods, such as multivariable logistic or Cox proportional hazards analysis. It has been used to calculate the continuous probability of an event of interest, based entirely on the disease characteristics of an individual, without averaging or combining within a category (8). Currently, nomograms are being widely used in the prognosis of cancer and other specialized diseases to help clinicians make important treatment decisions (9). Compared to previous prediction models, nomograms are more accurate and have better performance characteristics (10). Furthermore, a nomogram does not require imaging or other precise measures to be interpreted and to predict functional outcome. Therefore, for busy clinicians, the nomogram is an easier method for predicting functional outcomes in routine practice.

This study aimed to set up a clinical predictive score system to distinguish KD patients from non-KD febrile infection controls.

## Methods

### Study Participants

This study was based in the Shenzhen Baoan Women's and Children's Hospital in China and took place between August 2016 and July 2019. We enrolled KD children who had a fever for more than three days (38°C ear temperature), were <10 years old, and who had not undergone any IVIG or steroid therapy in the past one month. Anyone with a history of autoimmune diseases or congenital cardiovascular diseases was excluded. Age- and gender- matched febrile illness patients were also enrolled, including those with bronchopneumonia or pneumonia (80.5%), bronchitis (8.6%), upper respiratory infection (2.7), sepsis (2%), and other febrile diseases (1%, including enteritis, encephalitis, and urinary tract infection). The clinical diagnostic criteria of KD were based on the revised 2017 American Heart Association (AHA) diagnostic criteria for KD (11).

### Ethical Approval and Consent to Participate

We obtained informed consent from the parents or guardians of all subjects prior to enrolling patients in the study. The study was conducted pursuant to the Declaration of Helsinki. The Institutional Review Board of Shenzhen Baoan Women's and Children's Hospital, Shenzhen, China approved this study (IRB No. LLSCHY2019-07-01-01).

### Data Collection

Demographic data including information on gender, age, and body weight of the enrolled children were recorded, as were the following laboratory examination results: white blood cell count (WBC), neutrophil percentage, lymphocyte percentage, hemoglobin, platelet count (PLT), eosinophil percentage, mononuclear cell percentage, C reactive protein (CRP), procalcitonin, alanine aminotransferase (ALT), aspartate aminotransferase (AST), albumin, and erythrocyte sedimentation rate (ESR) for further analysis. No data were missing. Authors were not able to identify participants from this information during and after the study.

### Statistical Analysis

We used SPSS13.0 statistical software for analysis. The nomogram was developed based on the R software (Math Soft, Cambridge, Massachusetts). Mean ± standard deviation (X̄ ± s) was used for measurement data, while n and percentage were used for enumeration data. Normal distribution data were compared using an independent sample *t*-test or single factor analysis of variance. We adopted the rank sum test to compare non-normal distribution data. We also carried out the chi-squared test, and *p*-values < 0.05 were considered statistically significant. Multivariable logistic regression was used to analyze factors that influenced KD. We converted continuous data into classified data according to the data cutoff value of the largest area under the receiver operating characteristic curve (ROC) for each purpose. Furthermore, we created the nomogram using the results of the logistic regression equation. Hosmer and Lemeshow were used to determine whether the logistic prediction equation was suitable. We also assessed the performance of the nomogram using discrimination and calibration and calculated the area under the receiver operating characteristics (AUC-ROC) curve to assess the discrimination capacity of the model (12). The calibration of the models could be assessed using calibration plots, which can predict probabilities against actual observed risk (13).

## Results

We enrolled a total of 800 cases in this study, with an average age of 25.5 ± 19.2 months, including 485 males (60.6%), 315 females (49.4%), 249 KD patients (31.1%), and 551 non-KD febrile infection illness cases in the control group (68.9%).

### Selected Model Factors

We observed no statistical difference in body weight under univariate analysis (*p* > 0.05). However, WBC, neutrophil percentage, lymphocyte percentage, eosinophil percentage, monocyte percentage, hemoglobin, platelet, CRP, procalcitonin, ALT, AST, albumin, and ESR all demonstrated significant differences (*P* < 0.05), as shown in Table 1.

**Table 1:** Demographic data of the Kawasaki disease and non-KD febrile infection illness groups.

**Group**	**KD**	**Non-KD febrile infection illness**	***p*-value**
Male gender (%)	61.4%	60.3%	0.75
Age (month)	25.9 ± 19.4	25.3 ± 19.1	0.68
Body weight (Kg)	11.9 ± 3.8	13.8 ± 3.8	0.44
WBC (x10^9^/L)	14.3 ± 5.8	10.4 ± 5.3	<0.001
Hemoglobin (g/L)	107.8 ± 10.5	113.3 ± 12.3	<0.001
Platelet (x10^9^/L)	366.8 ± 138.6	316.3 ± 131.1	0.001
Neutrophil percentage	60.3 ± 17.2	45.8 ± 27.5	0.001
Lymphocyte percentage	29.5 ± 15.7	43.9 ± 17	0.001
Mononuclear percentage	7.8 ± 3.5	10.2 ± 5.7	<0.001
Eosinophil percentage	2.5 ± 2.6	0.9 ± 1.7	<0.001
CRP (mg/L)	177.6 ± 58.9	26.9 ± 33.6	<0.001
ALB (g/L)	37 ± 5.3	40.8 ± 4.1	<0.001
ALT (U/L)	57.5 ± 100.9	22.9 ± 29.9	<0.001
AST (U/L)	65.9 ± 111	50.9 ± 49.3	0.001
PCT (ng/L)	1.7 ± 3.4	0.86 ± 4.9	0.023

*KD, Kawasaki disease; WBC, white blood cell count; PLT, platelet count; CRP, C-reactive protein; PCT, procalcitonin; ALT, alanine aminotransferase; AST, aspartate aminotransferase; ALB, albumin; ESR, erythrocyte sedimentation rate*.

### Predictive Nomogram for the Probability of KD

**Figure 1:**
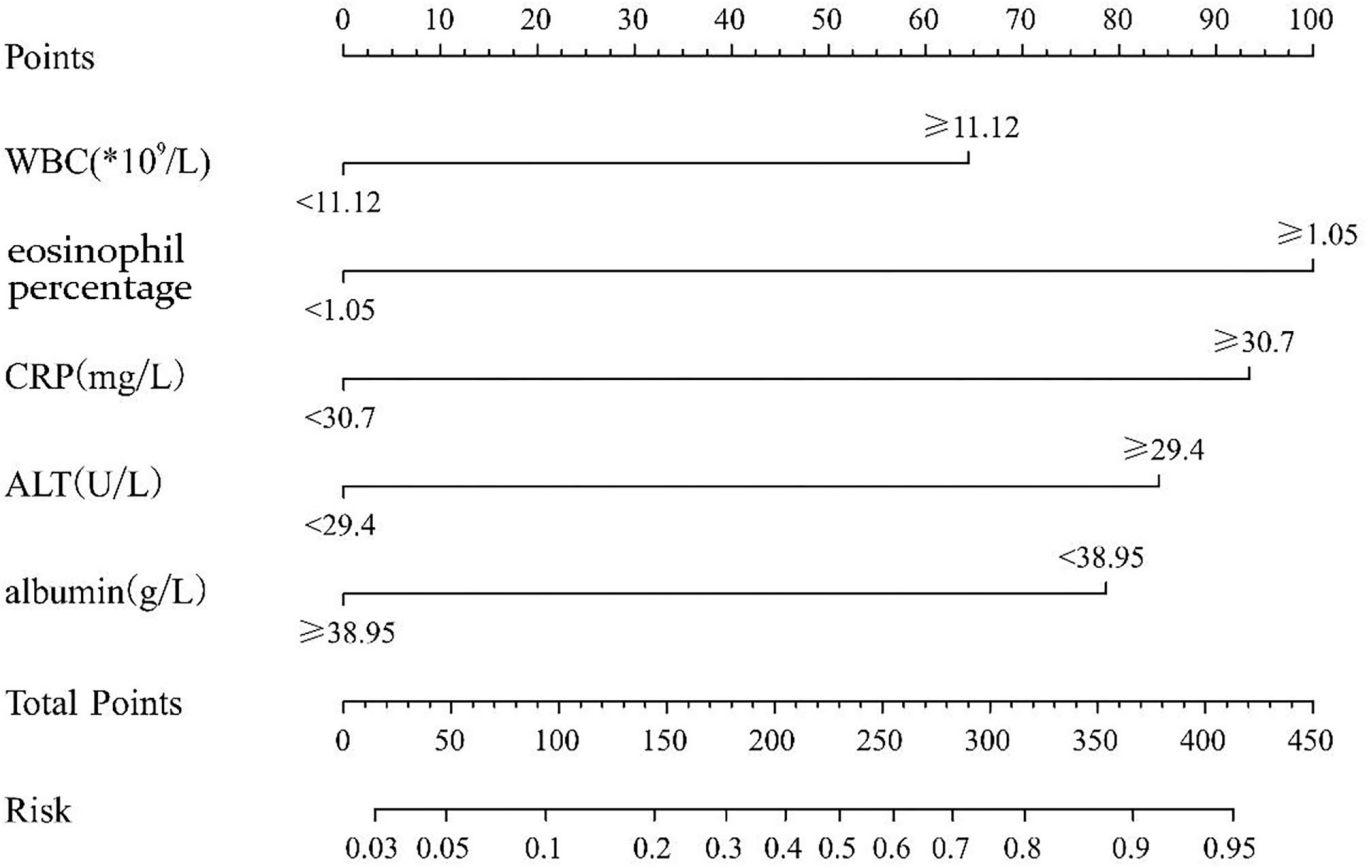
The nomogram prediction score of Kawasaki disease in the differentiation of non-Kawasaki disease febrile infection illnesses (WBC, white blood cell count; CRP, C reactive protein; ALT, alanine aminotransferase). The results from the multivariable regression analyses were used to construct the nomograms that predicted Kawasaki disease. A score proportional to the log of the odds ratio was assigned to each independent predictor. The total score for each case was assigned by drawing a vertical line from the appropriate point for each predictor down to the score scale, and summing these scores.

We randomly divided the 800 participant children into either the modeling group (70%) or the validation group (30%) using a process previously described in another report (14). Of those, 562 cases were in the modeling group, including 178 KD children (31.6%) and 384 cases of non-KD febrile infection, and 238 cases were in the validation group, including 71 KD children (29.8%), and 167 cases of non-KD febrile infection illness. An appropriate cutoff value was selected using the ROC curve, and multiple logistic regression analysis was performed on the modeling group. As shown in Table 2, the statistical results demonstrated that WBC, eosinophil percentage, albumin, ALT, and CRP were independent risk factors for differentiating KD from febrile infection illness. The nomogram was created according to the logistic regression results of the modeling group, as shown in Figure 1 (15). In the prediction model, high eosinophil percentage is the best predictor of KD (100 points), followed by high CRP (93 points), high ALT (84 points), low ALB (79 points), and high WBC (64 points). The total score is 420, and the probability of KD occurrence of each corresponding score is shown in Table 3. The total score can be easily calculated by adding together all the individual scores. Through the total score analysis and reflection of the lower total point scale, we were able to estimate the probability of KD and distinguish it from other febrile infection illnesses.

**Table 2:** Multivariable logistic regression analysis of Kawasaki disease and non-KD febrile infection illnesses.

	**Sig**.	**OR**	**95% C.I. for OR**	
			**Lower**	**Upper**
WBC(≥11.12*10^9^/L, <11.12*10^9^/L)	<0.001	2.826	1.723	4.637
Eosinophil percentage (≥1.05%, <1.05%)	<0.001	5.015	3.068	8.197
CRP (≥30.7 mg/L, <30.7 mg/L)	<0.001	4.510	2.727	7.456
ALT (≥29.41 U/L, <29.40 U/L)	<0.001	3.881	2.193	6.869
ALB (≥38.95 g/L, <38.95 g/L)	<0.001	3.554	2.183	5.784
Constant	<0.001	0.024		

*OR, odds ratio; CI, confidence interval, WBC, white blood cell count; CRP, C-reactive protein; ALT, alanine aminotransferase; ALB, albumin*.

**Table 3:** The incidence risk of Kawasaki disease corresponding to the total score.

**Total points**	**Kawasaki disease risk prediction (%)**
15	0.03
48	0.05
94	0.10
144	0.20
178	0.30
205	0.40
230	0.50
255	0.60
283	0.70
316	0.80
366	0.90
413	0.95

### 3.3 Performance of the Nomogram

**Figure 2:**
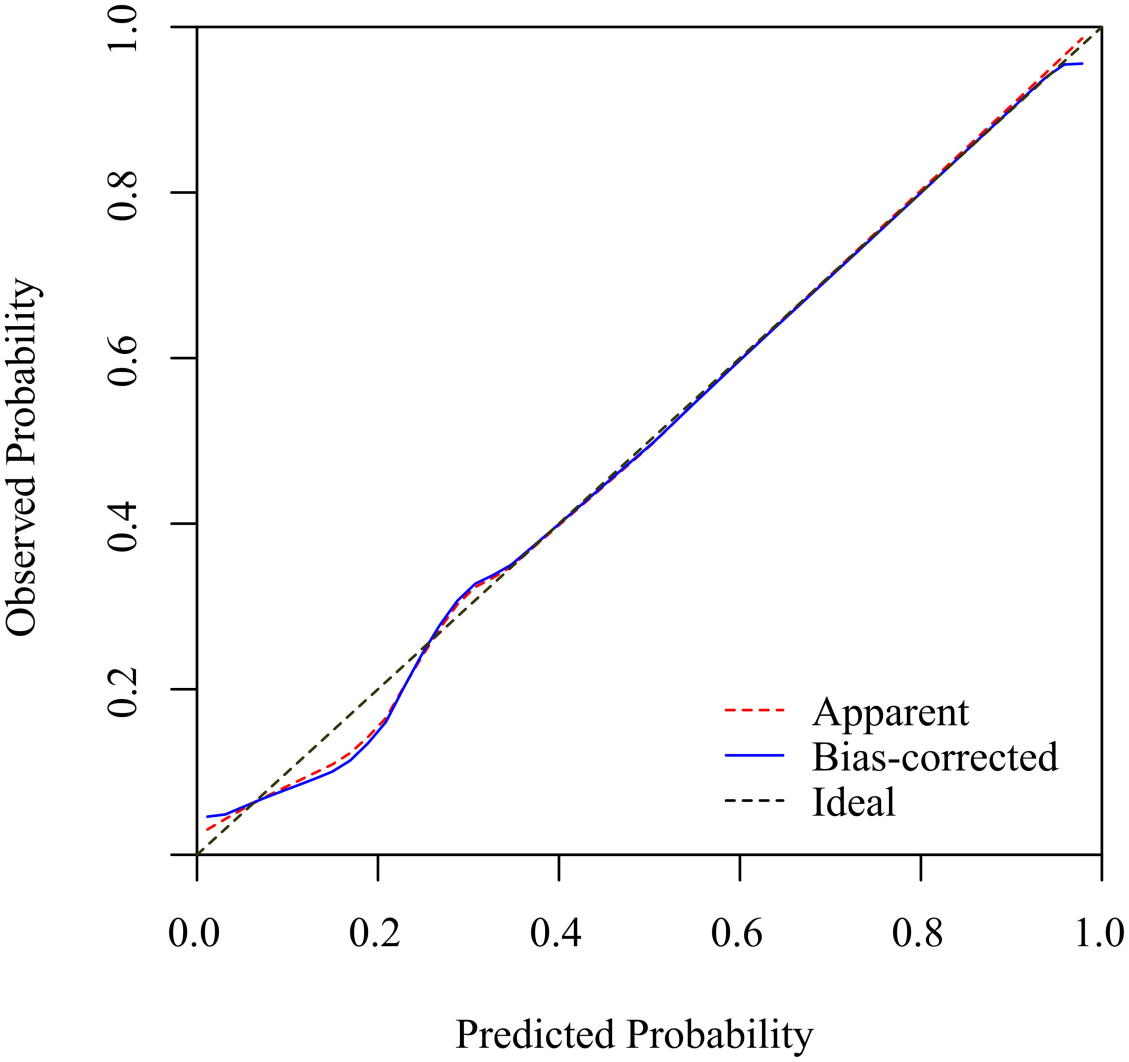
The calibration curves for the nomogram. The x-axis represents the nomogram-predicted probability, while the y-axis represents the actual probability of KD. A perfect prediction would correspond to the 45° dashed black line. The dotted red line represents the entire cohort (*n* = 238), and the solid blue line is bias-corrected by bootstrapping (B = 1000 repetitions), representing the observed nomogram performance.

Based on the receiver operating characteristic analysis, the nomogram showed good discrimination, with an area under the ROC of 0.873 (95% confidence interval, CI: 0.839–0.907) in the modeling group and 0.905 (95% CI: 0.862–0.948) in the validation group. The sensitivity and specificity were 75 and 89.1%, respectively, in the model group and 84.1 and 86% in the validation group. Figure 2 shows a calibration curve of the nomogram, indicating that the KD probabilities predicted by the nomogram agreed with the actual probabilities. The calibration curves for the KD outcome demonstrated no apparent departure from fit, with good correspondence between the predicted and the actual outcome. This study consisted of 104 KD patients with CAL data available for analysis (70 without CAL and 34 with CAL). We observed no significant difference regarding CAL formation (*p* = 0.93) when it was analyzed using this nomogram model. Furthermore, 173 KD patients with IVIG data were available for analysis (12 with IVIG resistance and 161 with IVIG responsiveness). A significantly higher percentage of more than 50% KD risk were found in the IVIG-resistance group (100 vs. 73%, *p* = 0.03).

### Independent Cohort for Validation

An independent cohort of 200 KD and 200 febrile controls from Kaohsiung Chang Gung Memorial Hospital, Taiwan were enrolled for validation. There were significant difference in WBC (13.4 ± 0.34 vs. 10.5 ± 0.43 × 10^9^/L, *p* < 0.001), eosinophil percentage (3.2 ± 0.22 vs. 0.9 ± 0.12, *p* < 0.001), CRP levels (99.2 ± 5.8 vs. 29.9 ± 3.1 mg/L, *p* < 0.001), and ALT (82.9 ± 7.9 vs. 32.2 ± 3.4 U/L, *p* < 0.001) when compared KD with febrile controls. A significant difference was also found when using the cut-off values of nomogram in the validation cohort, including WBC (11.12^*^10^9^/L), eosinophil percentage (1.05 %), CRP (30.7 mg/L), and ALT (29.41 U/L) (all *p* < 0.001). There were no albumin levels available in the febrile controls of the validation cohort.

## Discussion

As a febrile disease primarily occurring in children under 5 years old, the pathological mechanism of KD is an immune-mediated systemic vascular inflammatory change. Although self-limited, KD has a high incidence of coronary artery damage, generally caused by coronary artery dilatation, stenosis, and even atretic rupture (16). At present, physicians diagnose KD mainly by using clinical manifestations, laboratory results, and echocardiography. No specific laboratory tests are currently available to distinguish KD from febrile infection illnesses. Tests and methods for early KD diagnostics are missing, but the timely diagnosis and treatment of KD is vital for preventing the long-term sequela of CAL. Although changes in laboratory indicators are not specific, some indicators still have great value with regard to differentiating KD. In this study, we used univariate and multivariable logistic regression, ROC curve, and nomogram to establish a novel prediction score system with WBC, CRP, albumin, ALT, and eosinophil, among which eosinophil had the highest weight point for differentiating KD from other febrile infection illnesses.

In many previous reports, the peripheral blood WBC count of KD children during the acute stage significantly increased, as did the CRP plasma levels, but these changes cannot effectively distinguish KD from other febrile illnesses for more than 3 days. Some studies have reported that total WBC counts were significantly higher in KD children than in non-KD febrile illness cases (mostly from viral infection) (17). The total WBC of KD is higher than in viral fever children but lower than in bacterial fever children (18). WBC counts were higher in KD with delayed diagnosis and were associated with the left ventricular systolic function (17).

Many studies have reported that CRP is significantly increased in KD patients with coronary artery complications (CALs) compared to KD patients without CALs (19). Although the specificity of these two conventional inflammatory indicators (WBC, CRP) does not reach significance, they are commonly used in clinical practice and have more practical guiding significance for the clinical diagnosis of KD in the related AHA guidelines (20).

In our study, eosinophil percentage was the highest weighted score for the risk factor of KD, making it a crucial predictor in our novel nomogram prediction model. Elevated eosinophil in KD, which we have also found in our previous studies, indicated that eosinophilia was associated with IVIG-responsiveness and could prevent CAL formation (6, 21). Some data also showed that the eosinophils percentage and absolute eosinophil count were elevated in acute KD, as well as that the percentage of eosinophils continued to rise, peaking during the convalescent phase (22). Some studies have demonstrated that the incidence of eosinophilia in the peripheral blood of patients with incomplete KD is significantly higher than that of the KD group. In diagnosing incomplete KD, unexplained eosinophilia may be helpful (23). Although the underlying mechanism of increased eosinophils in KD is unclear, the accumulation of eosinophils in micro vessels and the increase of eosinophils in peripheral blood may be involved in the pathogenesis of KD (24). Taken together, eosinophil may play a protective role or have an anti-inflammatory effect in KD through the T-helper 2 cytokine (IL-4) (6, 25).

Lindsley et al. reported that eosinophils are circulating and that tissue-resident leukocytes have potent proinflammatory effects in many diseases. Recently, eosinophils have been shown to have various functions besides those related to allergies, including immunoregulation and antiviral activity. There are also some questions related to coronavirus disease 2019 (COVID-19) concerning eosinophils, which affect recommended prevention and care. Eosinopenia has been concluded to serve as a prognostic indicator for more severe COVID-19 and is likely a secondary phenomenon that does not directly contribute to the course of the disease (26).

In our study, reduced albumin is a predictor of the KD diagnostic model, but the mechanism of albumin reduction in KD children remains unclear. Dominguez et al. reported that the plasma levels of albumin were significantly lower in KD children than in febrile controls (27). Such decreased albumin levels may be related to increased vascular permeability, which is caused by the acute vascular inflammatory response of KD. Increased vascular permeability can cause the extravasation of endovascular substances, which may potentially be mediated by hormones, nerve innervation, or cytokines (especially il-2, interferon-alpha, and il-6) (28). The degree of decrease in albumin levels may reflect the severity of vascular inflammatory response. One study by Kuo et al. indicated that the lower the albumin level in KD children, the greater the risk of CAL (29).

We also found increased ALT to be an independent predictor for differentiating between KD and non-KD febrile illnesses. Most patients with elevated transaminase only demonstrated mild elevation, less than twice the normal upper limit. Elevated ALT is not an important cause of morbidity or mortality in KD patients but is a common finding during the acute phase of KD. Liver involvement ranges from the mild asymptomatic elevation of liver enzymes to severe cholestatic hepatitis and/or cholecystoid effusion (30). A US study identified ALT>60IU/L as a risk factor for IVIG non-responsiveness in KD (31). Previous studies have indicated that KD patients with abnormally elevated liver enzymes tend to have an increased proportion of CALs (32).

Taking into account previous studies on this subject, as explored in our literature review, the model described in the present study is the first prediction model to use a nomogram to distinguish KD from non-KD febrile infection illnesses. Compared to other clinical prediction tools or scoring systems, the nomogram has higher precision and optimal identification characteristics due to its continuous use of scales to calculate the continuous probability of a particular outcome (10). As a result, the nomogram provides superior personalized risk estimates that can contribute to modern medical decision-making.

This study has certain limitations. First, it is a single retrospective study and does not consider an entire country or multiple centers and thus may have selection bias. Second, the sample size is relatively small; however, the variables of the qualified patients enrolled are complete and correct.

## Conclusion

The present study demonstrates a novel prediction score system by using WBC, CRP, eosinophil percentage, albumin, and ALT to differentiate KD from other febrile infection illnesses. This study is the first to use a nomogram to develop a prediction model for KD, as well as to demonstrate the importance of eosinophil.

## Data Availability Statement

The raw data supporting the conclusions of this article will be made available by the authors, without undue reservation.

## Ethics Statement

The studies involving human participants were reviewed and approved by The Institutional Review Board of Baoan Maternal and Child Health Hospital, Shenzhen. Written informed consent to participate in this study was provided by the participants' legal guardian/next of kin.

## Author Contributions

X-PL, Y-SH, and H-CK conceptualized and designed the study and analyses and drafted and revised the manuscript. H-BX conceptualized and designed the study and analyses, participated in the design of the questionnaire, supervised all data analyses, and reviewed and revised the manuscript. YS participated in the design of the questionnaire, conducted analyses, and created the tables. X-LL, W-DH, Q-ZL, C-YL, and XL helped conceptualize this article, contributed to the interpretation of the findings, and reviewed and revised the manuscript. All authors participated in team discussions of data analyses, approved the final manuscript as submitted, and agree to be accountable for all aspects of the work.

## Conflict of Interest

The authors declare that the research was conducted in the absence of any commercial or financial relationships that could be construed as a potential conflict of interest.
